# Development of a district mental healthcare plan in Uganda

**DOI:** 10.1192/bjp.bp.114.153742

**Published:** 2016-01

**Authors:** Fred N. Kigozi, Dorothy Kizza, Juliet Nakku, Joshua Ssebunnya, Sheila Ndyanabangi, Blandina Nakiganda, Crick Lund, Vikram Patel

**Affiliations:** **Fred N. Kigozi**, MBChB, MMed, **Dorothy Kizza**, BASS, MSc, **Juliet Nakku**, MBChB, MMed, **Joshua Ssebunnya**, BSc, MSc, Butabika National Referral and Teaching Mental Hospital, Makerere University, Kampala, Uganda; **Sheila Ndyanabangi**, MBChB, MPH, Mental Health Division, Ministry of Health, Kampala, Uganda; **Blandina Nakiganda**, MBChB, MPH, Health Department, Kamuli District Local Government, Uganda; **Crick Lund**, BA, BSocSci, MA, MSocSci, PhD, Alan J Flisher Centre for Public Mental Health, Department of Psychiatry and Mental Health, University of Cape Town, South Africa, and Centre for Global Mental Health, Institute of Psychiatry, Psychology and Neuroscience, King's College London, UK; **Vikram Patel**, MRCPsych, PhD, FMedSci, Centre for Global Mental Health, London School of Hygiene and Tropical Medicine, UK, Public Health Foundation of India, New Delhi and Sangath, India

## Abstract

**Background**

Evidence is needed for the integration of mental health into primary care advocated by the national health sector strategic investment plan in Uganda.

**Aims**

To describe the processes of developing a district mental healthcare plan (MHCP) in rural Uganda that facilitates integration of mental health into primary care.

**Method**

Mixed methods using a situational analysis, qualitative studies, theory of change workshops and partial piloting of the plan at two levels informed the MHCP.

**Results**

A MHCP was developed with packages of care to facilitate integration at the organisational, facility and community levels of the district health system, including a specified human resource mix. The partial embedding period supports its practical application. Key barriers to scaling up the plan were identified.

**Conclusions**

A real-world plan for the district was developed with involvement of stakeholders. Pilot testing demonstrated its feasibility and implications for future scaling up.

Neuropsychiatric disorders are estimated to constitute about 14% of the global burden of disease, with approximately 80% of people with mental illness living in low- and middle-income countries (LMIC) such as Uganda.^1,2^ Further, Uganda's history over the past five decades has been that of violence, with civil strife and wars resulting in high prevalence of depression and post-traumatic disorders, and high alcohol consumption.^3^ In recognition of the high burden of mental illness in Uganda, mental health has been given policy priority and is one of the components of the national minimum healthcare package over the past decade.^4^ The current national health policy and health sector strategic investment plan (2010–2015) has retained mental health among the components of the national minimum healthcare packages to be delivered at all levels of the health system and to be integrated into primary healthcare so as to improve access.^5^ Despite this enabling national policy framework, there are glaring gaps in mental health service delivery, especially at primary care level,^6^ and an absence of district plans.

Against this background, as part of the work of the PRogramme for Improving Mental health carE (PRIME),^7^ we aimed to describe the process of developing a feasible district mental healthcare plan (MHCP), and describe its enabling intervention packages and components to be delivered at the various levels in a district demonstration site with limited resources. This plan thus seeks to operationalise the national health policy framework at the service delivery level.

The objectives of the MHCP are to: (a) provide equitable access to high-quality evidence-based mental healthcare to the people of Kamuli district; (b) strengthen the engagement of communities in better mental healthcare; (c) promote the integration of mental health into primary healthcare and bridge the treatment gap; (d) promote and protect the human rights of people with mental, neurological and substance use disorders in the district; (e) change the negative attitudes and misperceptions of the population regarding these disorders, through community sensitisation; and (f) strengthen the workforce for these disorders in the district.

## Method

### Setting

Kamuli, the implementation district for PRIME, is a typical rural district in Eastern Uganda, with a population of approximately 500 800 people.^8^ The district has a fertility rate of 6.7, higher than the national average of 6.2 children per woman; a maternal mortality rate of 347 per 100 000 live births, compared with the national average of 438 per 100 000 live births and an infant mortality rate of 79 per 1000 live births, compared with the national average of 54 per 1000 live births.^9–11^ The district was chosen out of the 112 districts in Uganda (a country with a population of 35.4 million people)^11^ mainly because it was typical of many rural districts in the country, with inadequate staffing and limited mental health service provision at primary care level. It could therefore form a model district if assisted to develop and operationalise its MHCP. Kamuli is a peaceful district and was not affected by insurgence in Northern Uganda. Administratively, the district is made up of two counties, ten subcounties and a number of parishes and villages (online Fig. DS1).

Like all other districts, healthcare in Kamuli is offered at five levels of care, in line with the national policy.

- At the community level (health centre I), there are community health workers, also known as village health teams, who serve as a link between the community and health facilities. They are members of the village, selected to carry out health promotion and prevention activities.
- At the parish level are health centre II facilities. These are outpatient units staffed by nurses, offering care for common ailments such as malaria, pneumonia and wound care. There are 22 of these units in the district.
- At the subcounty level there are ten health centres at level III. These are headed up by physician assistants (clinical officers) and have day services as well as maternity units.
- At the county level are two health subdistricts (health centre IV facilities), each headed up by a general doctor. At this level, there are admission facilities and a theatre for Caesarean section and other minor surgical interventions.
- At the district level, there is one general public hospital and one private hospital, with a number of doctors, nurses and midwives among other staff; and the two serve as the referral centres for the district.^12^

### Developing the plan

The process of developing the MHCP for the district was the joint effort of the Uganda PRIME team and Kamuli District stakeholders; a mix of both men and women, including 9 health managers, 6 political leaders, 37 health workers, 10 patients, 14 carers and 21 lay people. First, a situational analysis of the district healthcare system was conducted to gain some insight into the organisation of the healthcare system and the available resources in the district.^12^ This was followed by formative research to gather views and perceptions of the stakeholders on provision and utilisation of mental healthcare in the district. Data were collected using a generic interview guide developed by the consortium with five broad themes: demand/access, delivery, recovery, accountability and stakeholder views on how the research generated by PRIME could be used for policy and practice in Uganda. Focus-group discussions and individual in-depth interviews were conducted with primary healthcare workers, community health workers/village health teams, people with mental health problems, other members of the community and members of the district administration and district health management team. These participants were purposively selected as key informants who had expertise in a particular area, or who represented key stakeholder groups for mental health in the district. In-depth interviews and focus-group discussions were facilitated by two PRIME project staff members (the coordinator and research officer), who are clinical psychologists with Masters degrees in clinical psychology. These facilitators were employed by Makerere University in Kampala, and visited the Kamuli district for the purpose of data collection. In total, 14 focus-group discussions, with an average of 7 participants per focus-group discussion (n = 84) and 13 in-depth interviews were conducted (n = 13). The focus-group discussions and in-depth interviews were transcribed verbatim and those in the local language (Luganda) were translated into English. Data were coded with the help of NVivo9, a qualitative data analysis package and analysed using a content analysis framework.^13^

Phase two involved the development of the theory of change (ToC) map for the MHCP. ToC is the articulation of the underlying beliefs and assumptions that guide a service delivery strategy and are believed to be critical for producing change and improvement.^14,15^ A generic PRIME ToC map was initially developed in consultation with the partners in all PRIME countries sites.^16^ Equipped with baseline information from the situational analysis and findings from the formative research, the PRIME Uganda team adapted the generic PRIME ToC map to the situation in Kamuli. This was done by conducting two ToC workshops with various stakeholders, including 4 administrators, 8 health managers and 14 health service providers.^17^ These were some of the stakeholders who had earlier participated in the in-depth interviews and focus-group discussions. The purpose of the first workshop was to orient the participants in doing ToC mapping, and to agree on feasible outcomes, indicators of progress and strategies for the MHCP that would lead to the broad programme goals of improving health, social and economic outcomes of people with PRIME-prioritised mental disorders: depression (including maternal depression), alcohol use disorders, psychosis and epilepsy. A ToC map was subsequently drafted based on data from the formative research and the ToC workshops with the guidance of PRIME members. This map was systematically reviewed and finalised in the second workshop. The rationale for using ToC methodology is that it ensures a theory-driven approach to the development, evaluation and implementation of interventions.^17^

The MHCP was subsequently developed using the generated ToC map that has been published elsewhere in this supplement.^16^ Through an iterative consultation process, the PRIME team worked with the district health management team to translate information in the ToC map into five packages of care earlier agreed on in the consortium. These packages include: awareness and knowledge enhancement, detection, treatment, recovery and programme management. For each of the packages, strategic objectives, activities, roles and responsibilities as well as indicators of progress were developed for each of the three different levels of care (i.e. organisational, health facility and community levels). The human resources (cadre and numbers) to implement the plan was derived from the situational analysis report and based on the existing staff establishment.

### Costing the plan

The plan was costed using the World Health Organization (WHO) Mental Health Gap Action Programme (mhGAP) costing tool, initially developed to estimate the cost of implementing and scaling up the core intervention of the mhGAP intervention guide,^18^ and subsequently adapted for use in PRIME. More details of the tool are provided by Chisholm *et al* in this supplement.^19^ For the purposes of the Kamuli MHCP, the aim of the tool was to estimate the overall resource needs and cost implications of a scaled-up package of mental healthcare in Uganda. In developing the plan, the resources required to implement it were identified, estimated and fed into the mhGAP costing tool for each of the priority disorders in order to determine the cost of implementation and scaling up.

### Piloting the MHCP

The plan was piloted between August 2013 and February 2014 to evaluate its feasibility and identify any possible challenges before rolling it out to the entire district. This was done at two healthcare delivery levels (organisational and health facility levels); covering four out of the five packages. At organisational level, the piloting exercise covered two packages: awareness raising and programme management. At facility level, the packages covered included awareness and knowledge enhancement, detection and treatment. Evaluation was done at the health facility level, using both qualitative and quantitative methods, and covering 12 out of the 34 health facilities. The focus of the evaluation was on training satisfaction and ability to identify the priority conditions. Training was conducted using the mhGAP intervention guide training materials.^18^ The mhGAP intervention guide is a clinical guideline designed for use by primary care practitioners, with algorithms to assist diagnosis and treatment of eight priority conditions.^20^

The first 25 health workers to be trained were followed up 3 months later in the field and subjected to the mhGAP self-reported competence questionnaire (http://bit.ly/competencequest). This questionnaire is administered to health workers (prescribers) before and after receiving training in the mhGAP intervention guide. The questionnaire assesses nine self-reported competence areas: diagnosis, management of emergencies, prescription, monitoring, follow-up, provision of advice about the condition, referral, improving individual/community access to treatment and provision of psychological support. Only 17 of them participated in this evaluation exercise, as the remaining 8 were reportedly away from their duty stations at the time of this evaluation. The number of health workers who reported improvement in the core areas is as follows: diagnosis (15), management of emergencies (8), prescription of antidepressants (16), monitoring (14), follow-up (16), provision of advice about the condition (16), referral (16), improving individual access to treatment (14) and provision of psychological support (15). In addition, four in-depth interviews (two with clinical officers and two with nurses) and one focus-group discussion (with primary healthcare nurses) were also held for feedback and assessing the impact of the training on the health workers' practice. Finally, health management information system (HMIS) records from 13 health facilities were reviewed with a particular focus on the mental health indicators, making a comparison of 6 months before and 6 months after the training, in order to be able to track the trend in detection and reporting for mental health conditions. The HMIS records the number of patients treated for the various conditions at the different health facilities. This information is sent to the district health office on a monthly basis. These records were utilised as this provides a sustainable source of data to continue to monitor MHCP implementation over time. The results of the piloting were shared with some of the key stakeholders in the district and used in the processing of refining the MHCP.

## Results

In this section, we present an overview of the district MHCP as developed from the processes described above, including the formative research, ToC workshops, development of the plan and piloting of the plan.

### The district MHCP

The MHCP that resulted from the process described above is organised into five packages of care, and delivered at three levels of health service delivery: health organisation/management level, health facility level and community level.

#### The health organisation level

This level deals mainly with administrative and management aspects of health services at the district level. The two packages of care covered at this level are programme management, and awareness and knowledge enhancement. The details of the components, objectives, the health providers and their roles are provided in Table 1.

**Table 1:** Packages of care, components, objectives and implementers of the mental healthcare plan at the organisation level

Package and component	Objective	Providers and their roles
1. Awareness and knowledge enhancement				
1.1 Engagement/advocacy/mental health literacy of stakeholders to increase awareness about mental, neurological and substance use disorders in the district	To increase the awareness of managers and important stakeholders on mental health issues including the impact of gender on mental health, in order to facilitate their involvement in mental health programmes (buy-in)	District health officer Roles: identifying and establishing correspondence with the target stakeholders District mental health focal person and PRogramme for Improving Mental health carE (PRIME) team Roles: organising and facilitating the awareness and sensitisation workshops/activities

2. Programme management				
2.1 Drug supply chain management	To ensure adequate drug supply for mental, neurological and substance use disorders in the district	District health officer Roles: secure mental, neurological and substance use disorder drugs; monitor stocks District mental health coordinator Roles: advocates for procurement of mental, neurological and substance use disorder drugs; monitors availability of the drugs for these disorders in health facilities; reports drug situation to district health officer
2.2 Health management information system (HMIS)	To ensure a functional gender disaggregated HMIS for mental, neurological and substance use disorders	HMIS records officer Roles: to compile, summarise and report data on mental, neurological and substance use disorders; to offer supportive supervision District mental health coordinator Roles: to monitor and supervise compilation of mental, neurological and substance use disorder data from facilities; to ensure that the data for these disorders are captured and submitted
2.3 Human resource support, motivation and supervision	Plan and coordinate human resource for management of mental, neurological and substance use disorders	Chief administrative officer Roles: recruit and deploy health workers for management of mental, neurological and substance use disorders (to be sensitive to gender balance); plan for human resource for these disorders; supervise health workers; plan for capacity building for health workers District health officer and mental health coordinator Roles: both to monitor deployment and attrition of human resource for mental, neurological and substance use disorders; both to report human resource needs to the chief administrative officer
2.4 Capacity building	To equip district trainers with skills to instruct and supervise health workers in the use of the World Health Organization Mental Health Gap Action Programme	National and regional mental health trainers Roles: train and supervise district trainers Mental health coordinator and PRIME team Roles: train and supervise trainees as they train other health workers
2.5 Routine monitoring and evaluation	To conduct ongoing management monitoring, evaluation and quality control	District health management team and PRIME team Roles: manage, monitor, evaluate and ensure quality of mental health services Community advisory boards Roles: monitor provision of services

#### The health facility level

The facility level comprises the primary healthcare facilities, where health services are delivered. The packages of care covered at this level include awareness and knowledge enhancement, detection, treatment and recovery. The components for each of these packages, together with their objectives, health providers and their roles, are presented in Table 2.

**Table 2:** Packages of care, components, objectives and implementers of the mental healthcare plan at health facility level

Package and component	Objective	Providers and their roles
1. Awareness and knowledge enhancement		
1.1 Standardised in-service training for primary healthcare workers using the World Health Organization Mental Health Gap Action Programme (mhGAP) intervention guide	To improve knowledge and skills of primary healthcare workers (including midwives) in detection and treatment of mental, neurological and substance use disorders To improve attitudes and reduce stigma of primary healthcare workers towards people with mental, neurological and substance use disorders	Mental health staff from within the district and the regional referral hospital Roles: train and supervise the general health workers and midwives in mental healthcare District health officer and PRogramme for Improving Mental health carE (PRIME) team Roles: organise training of the primary healthcare workers

2. Detection		
2.1 Screening and assessment	To screen and assess mental, neurological and substance use disorders among patients attending primary healthcare facilities using the mhGAP intervention guide	Primary healthcare nurses Midwives Medical clinical officers Roles: to assess patients for mental, neurological and substance use disorders Mental health staff from district and regional PRIME team Roles: all to participate in supervision of primary health workers

3. Treatment		
3.1 Psychotropic medication	To prescribe appropriate psychotropic medication for people with mental, neurological and substance use disorders	Primary healthcare nurses Midwives Medical clinical officers Roles: prescribing psychotropic drugs; monitoring the effects of the drugs; referring appropriately District health managers Roles: ensure constant supply of psychotropic medications Mental health staff from district and regional referral PRIME team Roles: regular supervision of primary health workers; monitoring availability of medications
3.2 Basic psychosocial support	To provide basic psychosocial support to people with mental, neurological and substance use disorders	Primary healthcare nurses Midwives Medical clinical officers Roles: to provide psychosocial support to patients with mental, neurological and substance use disorders including perinatal women; follow up clients; monitoring the effects of the drugs; referring appropriately Mental health staff from district and regional PRIME team Roles: regular supervision and monitor provision of care by primary health workers; monitoring primary healthcare workers

4. Recovery		
4.1 Continuing care	To provide follow-up and continuity of care to people with mental, neurological and substance use disorders in primary healthcare	Primary healthcare nurses Midwives Medical clinical officers Roles: follow-up care of patients Mental health workers at the district, regional level and PRIME team Roles: supervise and monitor provision of care of primary health workers; monitoring primary healthcare workers

#### The community level

This level concerns health interventions provided within the community. At this level, three packages of care are covered, including awareness and knowledge enhancement, detection and recovery. The components, objectives and providers of services, together with their responsibilities at this level, are detailed in Table 3.

**Table 3:** Packages of care, components, objectives and implementers of the mental healthcare plan at the community level

Package and component	Objective	Providers and their roles
1. Awareness and knowledge enhancement		
1.1 Community sensitisation and anti-stigma mobilisation	To raise community awareness about mental health and psychosocial problems to reduce stigma towards people with mental health problems in the community To increase support and demand for mental health services including perinatal mental health services	Village health teams Roles: sensitise the community on mental health issues Mental health coordinator Roles: supervision of village health teams Primary health workers, midwives, district health visitors Roles: sensitise the community on mental health issues
1.2 Training of village health teams	To train village health teams to identify and to offer basic psychosocial support to people with mental, neurological and substance use disorders in the community	Psychiatric clinical officers Mental health nurses in the district Roles: organise village health team training workshops; facilitate workshops National trainers and PRogramme for Improving Mental health carE (PRIME) team Roles: supervise the trainers

2. Detection		
2.1 Community detection	To increase the detection and referral of people with mental health problems within the community	Village health teams Roles: identify and support people with mental health problems in the community Family Community leaders Roles: identify and refer to village health teams/health facility

3. Recovery		
3.1 Outreach and adherence support	To ensure adherence to mental health treatments and provide continuing psychosocial support to people with mental illness	Primary healthcare nurses and midwives Village health teams Roles: conduct outreach and support patients in the community Mental health coordinators Roles: supervision of primary healthcare workers and monitoring of outreach activities
3.2 Community-based rehabilitation	To provide community-based rehabilitation to people with mental, neurological and substance use disorders	Basic Needs-Uganda Village health teams Roles: provide peer and livelihood support to people undergoing treatment for mental health problems Community development officers, extension workers Roles: to link people with mental illness to available community-based livelihood programmes

The human resources available to implement the MHCP were based on the existing establishment, include general medical doctors, clinical officers, nurses and midwives, among others. Details of the breakdown of numbers and cadres for those that were available at each health facility level are shown in Table 4.

**Table 4:** Health personnel available to implement the mental healthcare plan in Kamuli^a^

	Primary healthcare facilities				
Cadre	District hospital (100 beds)	Health centre IV (30 beds)	Health centre III (out-patient)	Health centre II (out-patient)	Health centre I
General doctors	2 (0.40)	2 (0.40)	–	–	–

Clinical officers	7 (1.40)	7 (1.40)	20 (3.99)	–	–

Nurses (general)	62 (12.38)	10 (2.00)	23 (4.59)	15 (3.00)	–

Nurse midwives	36 (7.19)	7 (1.40)	33 (6.59)	16 (3.19)	–

Nursing assistant	12 (2.40)	3 (0.60)	23 (4.59)	15 (3.00)	–

Psychiatric clinical officer	1 (0.20)	–	–	–	–

Psychiatric nurses	2 (0.40)	2 (0.40)	0	–	–

Community health workers	–	–	–	–	1408 (281.15)

Total	122 (24.36)	31 (6.19)	99 (19.77)	46 (9.19)	1408 (281.15)

a. Numbers are actual full-time equivalent (FTE) staff in the district, with the FTE per 100000 population in parentheses. There is 1 district hospital, 2 health centre level IV facilities, 10 health centre level III facilities and 22 health centre level II facilities.

### Impact of the training on patient detection and care

A review of the HMIS data was done with a particular focus on the mental health indicators, making a comparison of 6 months before the training and 6 months after the training. Nearly all health facilities registered an increase in the case detection and reporting of some mental, neurological and substance use disorders following the training in the mhGAP. For example, HMIS records of 13 health facilities reviewed indicated a notable increase in the number of patients treated for epilepsy from 561 to 838, for depression from 71 to 95, for schizophrenia from 18 to 24 and for alcohol use disorders from 20 to 58.

The qualitative follow-up in-depth interviews and focus-group discussions revealed that individual health workers were managing more cases and making fewer referrals to the district hospital. The evaluation revealed challenges in the acceptability of the plan by some health workers who did not consider it part of their responsibility as general health workers to manage mental health problems. Detection and management of depression was reported to be quite challenging as the patients often present with somatic symptoms, which may be more difficult to identify as symptoms of depression. These health workers were also concerned that the community was still largely unaware of the existence of services at the lower facilities and that alcohol-related problems were still not being seen as a clinical problem, with low levels of healthcare-seeking from people with alcohol use disorders. Health workers recommended a need for more work in the community towards improving healthcare-seeking behaviour. It was noted that in one of the health facilities there was too much restriction of the nursing staff, who are not allowed to provide consultation and prescription to patients. This apparently prevented the trained nurses from identifying and treating patients with mental disorders at that clinic.

The general health workers felt that the training was short and that booster training sessions needed to be organised to consolidate the acquired knowledge and skills. They also identified a need for frequent supportive supervision from mental health specialists to enhance their skills.

## Discussion

The MHCP for Kamuli demonstrates a practical and realistic process of engaging the stakeholders in translating the national mental health policy and programme into an implementable district plan within the available resources, towards the reduction of the mental health treatment gap. A plan developed through these processes can be replicated (with minimal modifications) irrespective of the social demographic differences, as it is premised on the available resources. The critical drivers of the MHCP in Kamuli district included among others a clinical officer specialised in mental health and six psychiatric nurses. Districts without these cadres would need to identify equivalent cadres to provide mentorship to the general health workers to adapt this plan. The process of developing the plan and the embedding period have given us opportunity to reflect on the strength and limitations of the plan, as well as the challenges and barriers towards the scaling up of the plan and future research.

### Strengths of the plan

This district MHCP has a number of strengths. First, a wide range of key stakeholders in the district were engaged in a logical manner to promote ownership and acceptability of the components of the plan, both of which are essential for the success of its implementation. Second, the contents of the plan were informed by a situational analysis and qualitative studies in the district, both of which improve its relevance to the local context.^12^ Similarly, the development of the MHCP provides the methods for translating national health policies and guidelines into implementable service delivery plans at the lower levels that can be replicated in other districts. This is in line with the government of Uganda national health policy and health sector strategic investment plan, both of which advocate for integrated service delivery at primary care level. Although the health sector strategic investment plan has for a long time prioritised mental health as one of the components of the national minimum healthcare package intended to be delivered at all levels of care, this is the first time that a MHCP for a district has been developed, which can be replicated elsewhere in the country to guide the delivery of mental healthcare. The national Ministry of Health is committed to adapting the lessons learned from the PRIME MHCP in Kamuli to other districts in the country.

### Limitations of the MHCP

We anticipate some challenges in the implementation of the Kamuli MHCP. First, Kamuli district has a limited health budget with no specific allocation to mental health. However, it is expe,cted that the initial implementation of the MHCP will draw on the existing resources. The strain on the budget will became more noticeable with the scale up of the plan, and the resulting increase in service utilisation by people with mental, neurological and substance use disorders, particularly for medicines and supportive supervision. Second, our formative study revealed high levels of stigma towards people with mental health problems in the district. This is likely to influence health-seeking behaviour and utilisation of the services as provided for in the plan. However, the community sensitisation component within the awareness and knowledge enhancement package has been developed to address this challenge and reduce stigma levels and improve service utilisation. Third, we need to acknowledge the limited conclusions that can be drawn from our self-report questionnaire, used to evaluate changes in competency of primary care workers. Although this MHCP is quite comprehensive, many of its components are yet to be implemented and evaluated rigorously. Further more rigorous evaluation is necessary to give a clear sense of which of these components are feasible and can be scaled up with the limited resources available. Furthermore, data on the human resources training aspect during the embedding phase were from a few health workers, selected from some health facilities, and may not be representative of other health workers and facilities in the district. The community-level package components are yet to be implemented, as these are intended to stimulate demand for services. The study therefore provides preliminary information and describes the local development of an intervention, not evidence of its impact on the needs of people with mental health problems.

### Challenges and barriers

The results of the 3-month pilot testing during the embedding period underpin the need to intensify efforts in two areas. First, sensitisation of communities about mental health problems and their management is required to improve health-seeking behaviour. This will be monitored using the number of people with mental illness attending primary healthcare facilities. Second, follow-up of the trained health workers with regular supportive supervision to take care of the specific health workers' knowledge and competence gaps is required on an ongoing basis. These areas are to be taken into account during the roll out of the plan to the entire district.

### Lessons learned

There is a significant lack of published evidence on the ‘how’ of developing MHCPs that enable integration of mental health in primary care in LMIC. According to Saxena and colleagues, the only available similar studies on the African continent originate from South Africa, a middle-income country.^21^ Although many LMIC have enabling policies for the integration of mental health into primary healthcare, there is often a lack of translation of these policies into practice. This has been attributed to among other things: (a) lack of specialised mental health personnel to guide this process; (b) inadequate health infrastructure; (c) lack of capacity to provide psychological therapies; and (d) inadequate epidemiological data to enable policy makers to prioritise the integration of mental health at district level. This paper therefore provides the opportunity to document how contextually appropriate MHCPs can be developed and tested in a low-income country. Such a plan helps to translate policy into an implementable plan to address mental health needs in the district and can be adapted to other similar environments.

The health worker resources indicated in Table 4 is based on the current field staff establishment. This human resource mix for the MHCP implementation shows great reliance on the nursing and clinical officer levels, with a few general doctors and no specialist psychiatrists for the district. This implies that specialist supportive supervision will have to be provided by the regional referral hospital, in line with the health sector strategic and investment plan.^5^ Further, it is noted that the current human resource numbers are about half of the official staff establishment.^12^ It is expected that with buy-in and improved awareness of mental health needs at that management level of the district, more human resources will be recruited to improve service delivery. The implementation of the MHCP in Kamuli is required to work within the available resource constraints, with the introduction of new training, support and supervision as described above. The Kamuli plan is thus constrained by the national policy and strategic plan and the available human and financial resources, and unfortunately it was not possible for the Ministry of Health to provide additional human and financial resources in this context. Nevertheless, we believe that the available human resources, as outlined in Table 4, are adequate to implement the MHCP effectively, when provided with the required training, support and supervision.

### Future research

Future research should, among other areas, include evaluation of the implementation of the entire plan, including the benefits of integrated service delivery to provide evidence, particularly for policy makers, to enable them to make informed decisions on resource allocation.

The development of this plan was gradual and an iterative multiphased process that involved several key stakeholders, resulting in translation of the objectives of the national health sector strategic investment plan into an implementable programme-specific plan at district level. The plan was designed to address locally identified needs and gaps and to provide evidence-based interventions to respond to these needs for people with mental health problems. Although this work was within a rural district, it may be replicated in several other rural and peri-urban districts in the country. Evaluating its implementation in the real world will provide the necessary evidence for its replicability.
